# Efficacy of Microneedle Fractional Radiofrequency Combined With Platelet‐Rich Plasma for the Treatment of Melasma: A Split‐Face, Randomized Trial

**DOI:** 10.1111/jocd.70742

**Published:** 2026-02-13

**Authors:** Ziyan Chen, Yuhao Li, Yi Ou, Tingqiao Chen, Yangmei Chen, Jin Chen

**Affiliations:** ^1^ Department of Dermatology The First Affiliated Hospital of Chongqing Medical University Chongqing China

**Keywords:** melasma, microneedle fractional radiofrequency, platelet‐rich plasma, ultrasound

## Abstract

**Background:**

Melasma is a common facial pigmentary disorder that markedly impairs quality of life, yet current treatments often yield incomplete clearance and frequent relapse. Microneedle fractional radiofrequency (MFR) can remodel the dermis and enhance transdermal delivery, whereas platelet‐rich plasma (PRP) provides growth factors that may modulate melanogenesis and inflammation.

**Aims:**

To compare the efficacy and safety of MFR combined with PRP versus MFR alone in the treatment of facial melasma.

**Methods:**

In this prospective, randomized, evaluator‐blinded, split‐face trial, 30 patients with stable, symmetrical facial melasma received two sessions of MFR. One facial side was randomly assigned to receive adjunctive PRP and the contralateral side received saline. Outcomes included modified Melasma Area and Severity Index (mMASI) and hemi‐mMASI scores, VISIA skin analysis, patient‐reported satisfaction, and adverse events. Ultrasound was used to measure epidermal and dermal thickness at predefined facial landmarks.

**Results:**

Twenty‐nine patients completed the study. Overall mMASI and hemi‐mMASI scores decreased on both sides at the 3‐month follow‐up after the last treatment. VISIA analysis showed significant reductions in red area and brown spot scores on the combination side, whereas changes on the MFR‐alone side did not reach statistical significance. Ultrasound demonstrated increased dermal thickness at selected landmarks on each side, but the magnitude of change did not differ between sides.

**Conclusions:**

MFR is an effective and safe option for melasma in patients with Fitzpatrick skin types III–IV. Adjunctive PRP did not further improve global clinical pigment scores or ultrasound‐detected structural changes, but may offer added benefit for erythematous and pigmentary features detected by VISIA.

## 1 Introduction

Melasma is a common, multifactorial acquired hypermelanosis that typically presents as symmetrically distributed brown patches on the cheeks or forehead, most often affecting women with Fitzpatrick skin phototypes III–IV [1, 2]. Its pathogenesis is complex and involves increased local vascularity, chronic low‐grade inflammation, and disruption of the basement membrane and skin barrier, among other mechanisms [3, 4, 5, 6].

Because melasma preferentially affects exposed facial areas, it causes substantial psychosocial distress and impairs quality of life [1, 7]. Although multiple therapies are available—including topical depigmenting agents, chemical peels, oral tranexamic acid, and various energy‐based devices such as intense pulsed light and fractional lasers—management remains challenging, with high recurrence rates and treatment‐related adverse events, particularly post‐inflammatory hyperpigmentation in darker skin phototypes. Thus, safer and more durable treatment approaches are still needed [2, 8, 9, 10].

Microneedle fractional radiofrequency (MFR) has emerged as a promising modality for melasma and other pigmentary or textural disorders, delivering bipolar radiofrequency energy directly into the dermis to induce controlled thermal injury, modulate dermal vasculature, promote collagen remodeling, and help restore basement membrane integrity; the micro‐channels created also facilitate enhanced transdermal drug delivery [11, 12, 13]. Platelet‐rich plasma (PRP), an autologous concentrate of platelets, provides growth factors such as transforming growth factor‐β1 (TGF‐β1), which may suppress melanogenesis via downregulation of microphthalmia‐associated transcription factor (MITF)‐related pathways [14, 15]. Although both MFR and PRP have individually shown benefits in pigmentary disorders, randomized controlled data on their combined use for melasma remain limited, prompting us to conduct a prospective, randomized, split‐face clinical trial to compare MFR plus PRP with MFR alone in patients with stable facial melasma [16, 17].

## 2 Methods

### 2.1 Study Design

This was a monocentric, prospective, randomized, split‐face clinical trial with evaluator blinding. All procedures complied with the principles of the Declaration of Helsinki. Written informed consent was obtained from all participants prior to enrolment.

### 2.2 Population and Intervention

Patients were recruited from the Dermatology Department of the First Affiliated Hospital of Chongqing Medical University. Inclusion criteria were: age ≥ 18 years; clinically diagnosed facial melasma with a symmetrical distribution; and a stable disease phase, defined as the absence of melasma‐directed treatment and no subjective worsening or noticeable progression within the preceding 12 weeks, confirmed by clinical examination and Wood's lamp evaluation.

Exclusion criteria included: pregnancy or lactation; known photosensitivity; presence of systemic diseases that might interfere with wound healing; excessive exposure to sunlight or ultraviolet radiation within the past 3 months; or any treatment for melasma within the preceding 12 weeks.

Potential participants underwent a screening examination by dermatologists. Eligible patients who agreed to participate signed written informed consent forms.

### 2.3 MFR and PRP Therapy

An MFR device (Shenzhen Peninsula Medical Group, China) was used in this study. This system allows precise delivery of radiofrequency energy at multiple skin depths during a single needle insertion. The insertion depth can be adjusted from 0.5 to 3.5 mm, enabling treatment across 1 to 5 different tissue layers. PRP was prepared in the Department of Blood Transfusion, First Affiliated Hospital of Chongqing Medical University, using an automated apheresis system (blood component separator; continuous‐flow centrifugation) in a closed, sterile, single‐use tubing set. Briefly, autologous venous blood was processed with ACD‐A anticoagulant according to the manufacturer's programmed collection procedure, during which the device continuously separated blood components by centrifugation, selectively collected the platelet‐rich plasma fraction, and returned the remaining components to the patient. The collection was performed using a leukocyte‐reduction setting, yielding leukocyte‐poor PRP (LP‐PRP). PRP was administered intradermally at depths corresponding to the MFR‐induced microchannels to facilitate dermal diffusion. Before treatment, a topical lidocaine cream was applied to the entire face for 1 h to achieve local anesthesia. The skin was then thoroughly cleansed and disinfected with povidone‐iodine. MFR was performed on the full face.

Using a random‐number table, one side of each patient's face was assigned as the combination side (MFR + PRP) and the contralateral side as the MFR‐alone side. The MFR device was configured to deliver separate energy discharges in the superficial and deeper dermal layers, with treatment parameters selected based on facial anatomical considerations, melasma pathophysiology, and safety in Fitzpatrick skin types III–IV. Adjustable needle depths (0.8–2.5 mm) were chosen to primarily target the mid‐to‐deep dermis, where vascular proliferation, inflammation, and basement membrane disruption are known contributors to melasma, while minimizing excessive epidermal thermal injury and the risk of post‐inflammatory hyperpigmentation. The treatment settings were as follows: energy 4–8 W, pulse width 40–120 ms, and microneedle depth 0.8–2.5 mm. Needles were inserted vertically, and the treatment tip was kept in close contact with the skin. Each treated area was covered evenly with the RF electrode matrix; to ensure complete coverage, the RF matrices were allowed to overlap by approximately 20%.

Immediately after MFR, PRP was applied to the combination side, whereas physiological saline was applied to the MFR‐alone side. Cold compresses with medical cold patches were then applied to both sides.

Patients were instructed to keep the treated area dry for 72 h. After 72 h, regular skincare was permitted, and the use of moisturizers was recommended. Two identical MFR sessions were administered, with an interval of 2 months. During this interval, patients received one PRP injection on the combination side and one saline injection on the MFR‐alone side.

After the final MFR session, patients were followed monthly for 3 months (Weeks 12, 16, and 20 after baseline).

### 2.4 Evaluation

Standardized clinical photographs were obtained using the VISIA imaging system at Weeks 0, 4, 8, 12, 16, and 20 (corresponding to 3 months after the final treatment). Dermatoscopic images were captured at fixed anatomical landmarks for each patient at Week 0 and Week 20.

Ultrasound examinations were performed at Weeks 0 and 20. Ultrasound was used to measure the thickness of the epidermis, dermis, and subcutaneous fat layers on both sides at specific reference points (Landmarks A–C). Landmark A, at the midpoint of the forehead, was defined by the intersection of a vertical line drawn from the midpoint of the pupil and a horizontal line across the central forehead. Landmark B, at the masseter region, was identified by placing the probe parallel to the mandible, with its midpoint at the intersection of the masseter and the mandible. Landmark C was defined as the midpoint of the cheek.

All measurements were performed with the patient seated by the same experienced technician, who was blinded to the treatment allocation. The technician was instructed to apply minimal pressure with the probe to avoid compression of the skin.

The primary outcomes were changes in the modified Melasma Area and Severity Index (mMASI) and hemi‐mMASI scores between baseline and follow‐up. Two experienced dermatologists independently assessed mMASI and hemi‐mMASI scores in a blinded manner using standardized photographs to evaluate melasma severity and its change over time.

Safety assessments included the presence of erythema, edema, crusting, blistering, bleeding, post‐inflammatory hyperpigmentation, post‐inflammatory hypopigmentation, and scarring, which were recorded at each visit.

Subjective satisfaction was evaluated at the 2‐month follow‐up visit using a 4‐point scale ranging from 1 (very satisfied) to 4 (not satisfied).

### 2.5 Statistical Analysis

Statistical analysis was performed using SPSS Statistics software (version 26; IBM Corp., Armonk, NY, USA). Normally distributed continuous data are presented as mean ± standard deviation (SD), and non‐normally distributed data as median (min‐max). Paired *t*‐tests were used for normally distributed variables, and Wilcoxon signed‐rank tests were applied for non‐normally distributed paired data to compare outcomes between the two treatment sides. A *p* value < 0.05 was considered statistically significant. Due to the exploratory nature of this split‐face study, no formal a priori sample size or power calculation was performed. The sample size was determined based on feasibility.

## 3 Results

### 3.1 Patient Characteristics

**FIGURE 1:**
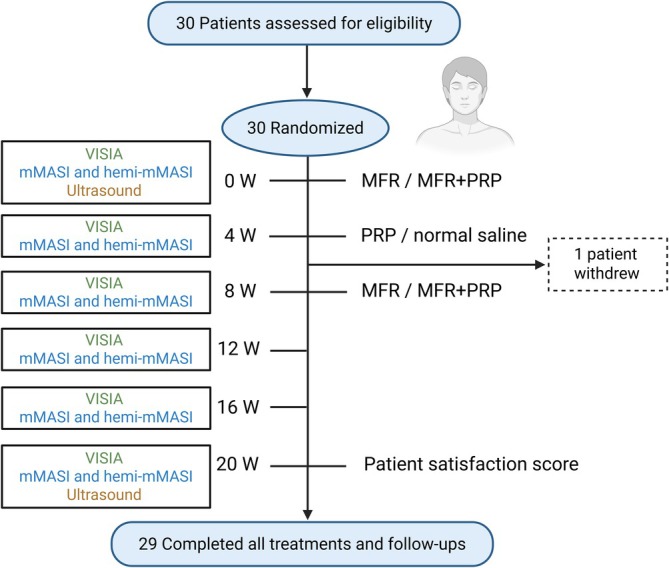
Flow diagram of patient enrolment and assessment schedule. Intermediate visits were scheduled for standardized photography and safety assessment; primary efficacy analyses were performed using baseline and end‐of‐follow‐up assessments.

A total of 30 patients were enrolled in the study. One patient withdrew, and 29 patients completed the treatment protocol and all follow‐up visits. Baseline demographic and clinical characteristics are summarized in Table 1. The mean age was 46.97 ± 7.05 years, and the mean duration of melasma was 12.34 ± 5.83 years. Twenty patients had Fitzpatrick skin type III and nine had type IV. Among the 29 patients who completed the study, 10 (34.45%) reported a history of previous treatments for melasma (Figure 1).

**TABLE 1:** Patient demographics.

Characteristics	Patients (*n* = 29)
Number of patients	29
Age	46.97 ± 7.05
Gender	
Female	28
Male	1
Duration of melasma	12.34 ± 5.83
Melasma family history	
Yes	
No	
Melasma treatment history	
Yes	10
No	19
MASI score	6.68 ± 3.58
Fitzpatrick skin types	
III	20
IV	9

### 3.2 Efficacy Outcomes

**FIGURE 2:**
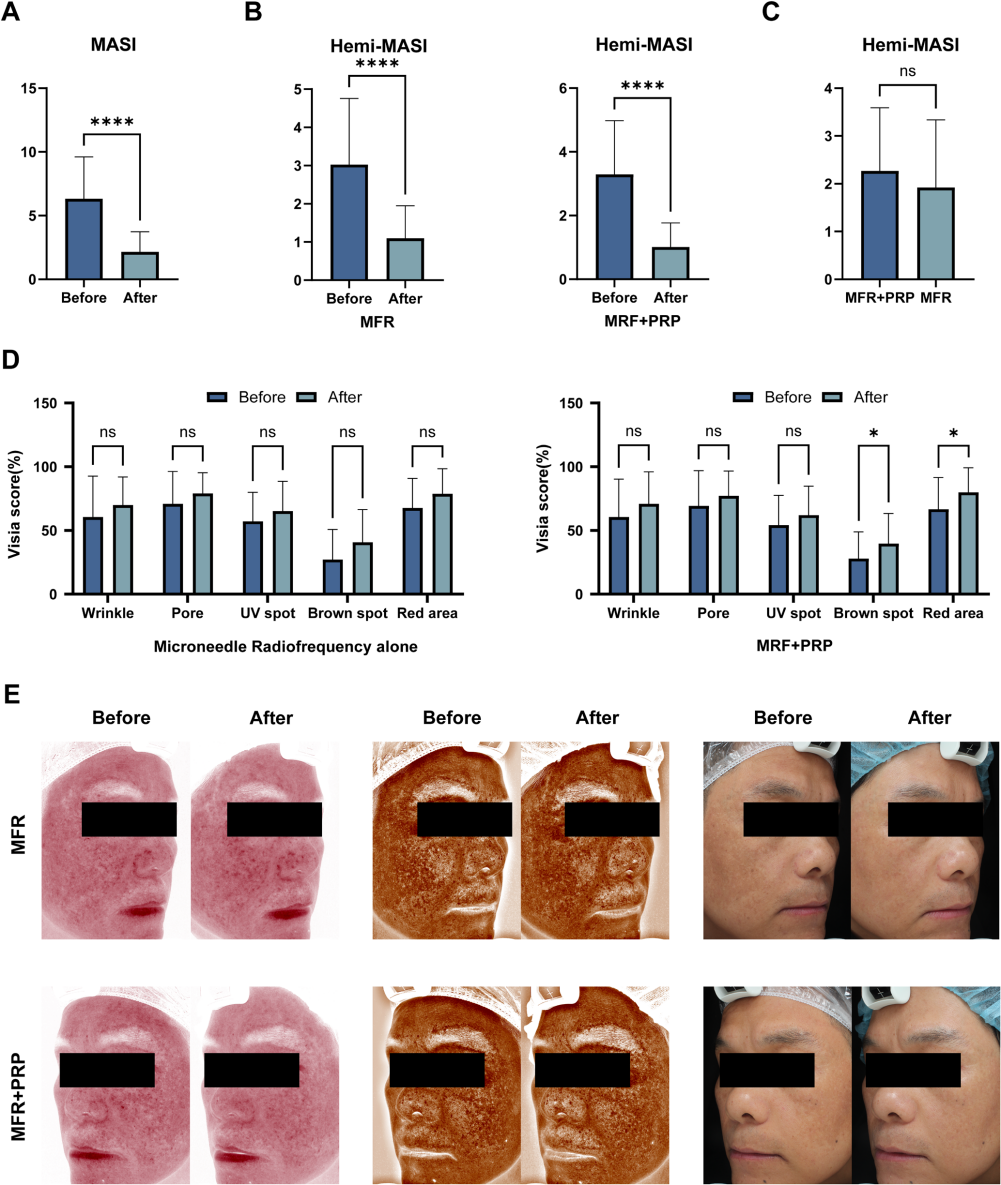
Changes in mMASI, hemi‐mMASI and VISIA parameters after treatment. (A) Overall mMASI scores from baseline to Week 20. (B) Hemi‐mMASI scores on the combination (MFR + PRP) and MFR‐alone sides. (C) Mean change in hemi‐mMASI from baseline, showing no significant between‐side difference. (D) VISIA red area, brown spot, wrinkle and UV spot scores at baseline and Week 20, with significant improvement in red areas and brown spots on the combination side only and non‐significant trends for wrinkles and UV spots. (E) Representative VISIA images at baseline and Week 20. ns, not significant; *p < 0.05; ****p < 0.0001.

Overall mMASI scores at Week 20 were lower than at baseline, indicating a reduction in global melasma severity (Figure 2A). Compared with baseline, hemi‐mMASI scores on both the combination and MFR‐alone sides decreased at every follow‐up visit (Figure 2B). However, there was no statistically significant difference between the two sides in the degree of hemi‐mMASI improvement (Figure 2C).

The VISIA skin analysis system was used to quantitatively assess red areas, brown spots, wrinkles, and UV spots at baseline and at the end of follow‐up. As shown in Figure 2D, red area and brown spot scores on the combination side improved significantly compared with baseline, whereas changes in these parameters on the MFR‐alone side did not reach statistical significance. Although significant within‐side improvements in VISIA red area and brown spot scores were observed on the MFR + PRP side, the between‐side comparison of change‐from‐baseline (Δ) values did not show statistically significant differences between the two treatments. After treatment, scores for wrinkles and UV spots showed a trend toward improvement on both sides, but these changes did not reach statistical significance (*p* > 0.05). Representative VISIA images from patients who completed the study are shown in Figure 2E.

### 3.3 Ultrasound Evaluation

At baseline, there were no statistically significant differences between the combination and MFR‐alone sides in the thickness of the epidermis, dermis, or subcutaneous fat at Landmarks A, B, or C (*p* > 0.05).

From baseline to Week 20, dermal thickness at Landmark A increased significantly on the combination side, and dermal thickness at Landmark C also increased significantly on the combination side. At Landmark B on the combination side, both epidermal and dermal thickness increased significantly.

**FIGURE 3:**
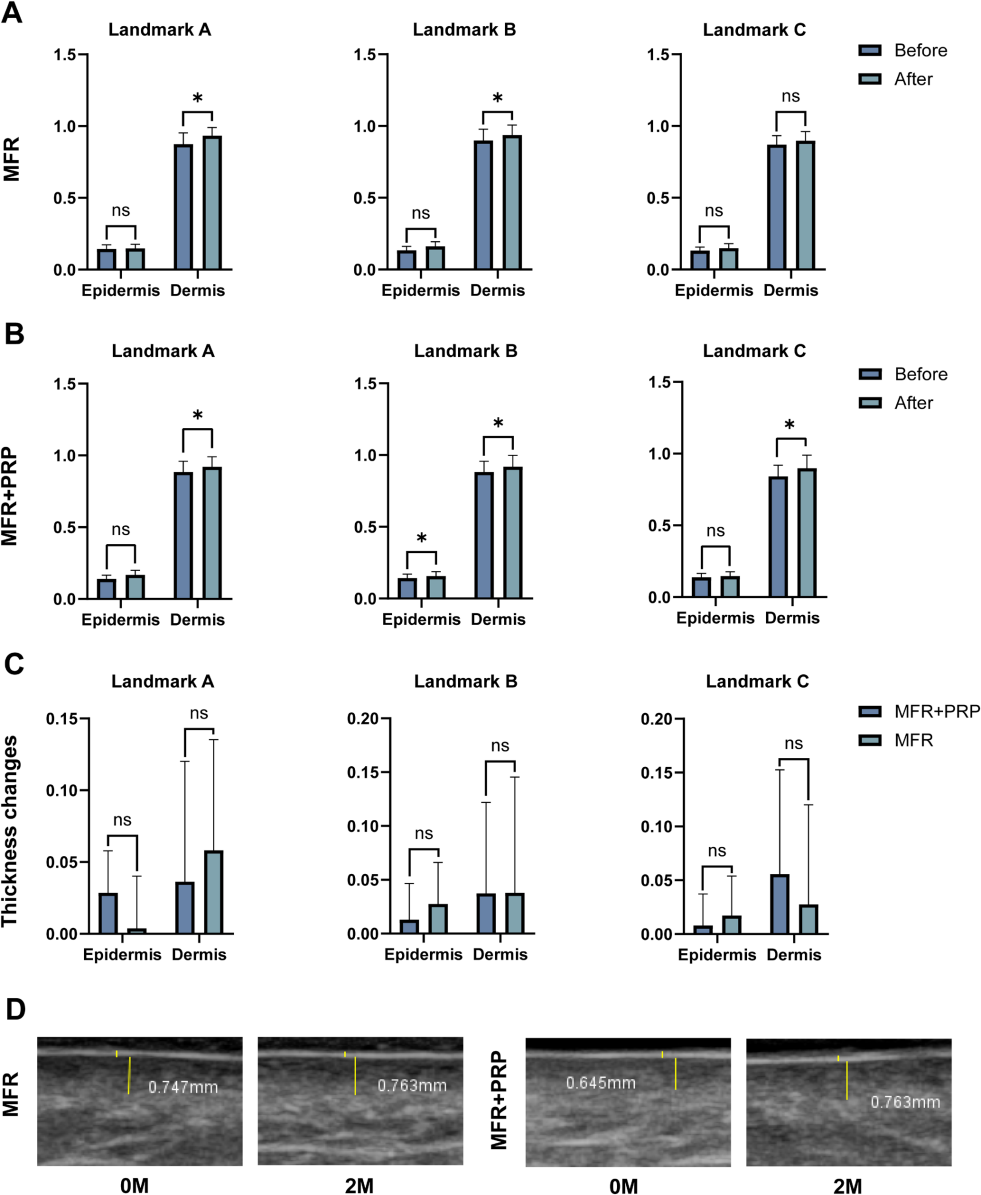
Ultrasound assessment of skin thickness at Landmarks A–C. (A) MFR‐alone side: epidermal and dermal thickness at Landmarks A–C before and after treatment. (B) MFR + PRP side: epidermal and dermal thickness at Landmarks A–C before and after treatment. (C) Quantitative comparison of thickness changes between the two sides at each landmark. (D) Representative ultrasound images of dermal thickness at baseline (0M) and Month 2 (2M) on the MFR and MFR + PRP sides. Epidermal and dermal thickness values are expressed in millimeters and represent averaged measurements obtained at predefined landmarks by a blinded technician. ns, not significant; *p < 0.05.

On the MFR‐alone side, dermal thickness increased significantly at Landmark A and at Landmark B between baseline and Week 20. However, when comparing the magnitude of thickness changes between the two sides at each landmark, no statistically significant differences were observed (Figure 3). These findings suggest that MFR itself played the key role in dermal remodeling detected by ultrasound, whereas adjunctive PRP did not confer an additional cumulative effect on ultrasound parameters.

### 3.4 Patient Satisfaction

**FIGURE 4:**
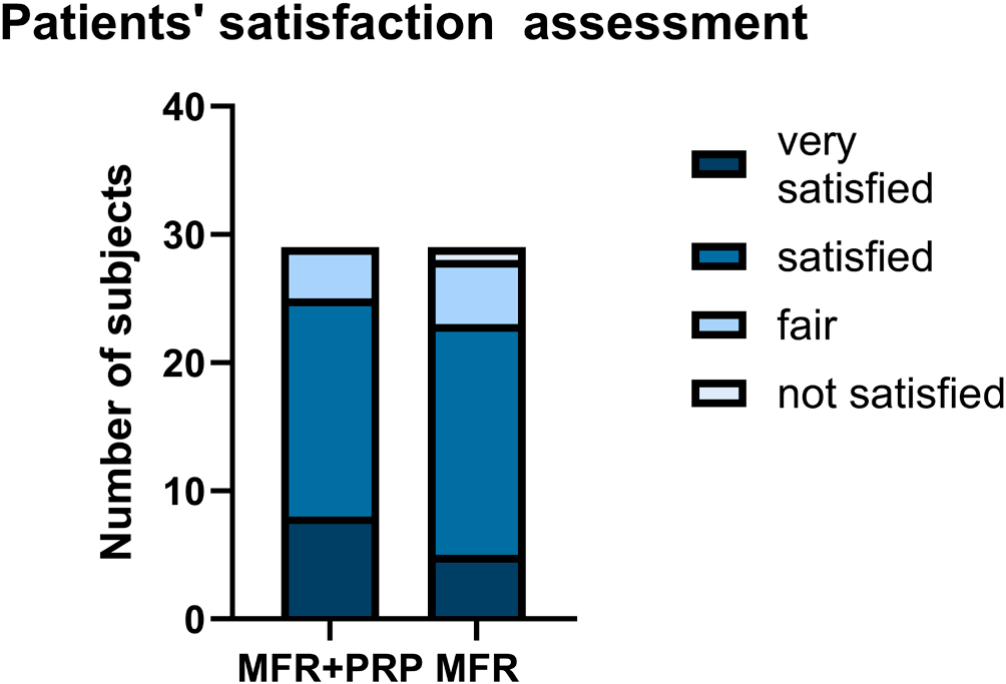
Patients' satisfaction assessment. Comparison of satisfaction levels between the MFR + PRP group and the MFR group. The stacked bar chart shows the number of subjects in each satisfaction category: Very satisfied, satisfied, fair, and not satisfied.

At the end of the study, 7 patients (24.13%) rated the improvement on the combination side as “very satisfactory,” and 5 patients (17.24%) rated the improvement on the MFR‐alone side as “very satisfactory.” Overall satisfaction scores did not differ significantly between the two sides (*p* > 0.05), indicating that patients perceived comparable subjective benefits from both treatment modalities (Figure 4).

### 3.5 Adverse Effects

The main side effects of MFR treatment were erythema, burning, and pain, which were generally mild to moderate in intensity. Burning and pain subsided within approximately 2 h, and erythema resolved within 3 days in all cases. Among the 29 patients, 3 reported facial dryness and pruritus approximately 2 weeks after treatment, which improved with routine skincare. No cases of blistering, scarring, post‐inflammatory hyperpigmentation, or hypopigmentation were observed during the treatment or follow‐up periods.

## 4 Discussion

In this prospective, randomized, split‐face trial, we compared MFR alone with MFR combined with PRP in patients with stable facial melasma using a combination of clinical scores, digital imaging, and ultrasound [18, 19, 20, 21]. Both treatment regimens led to significant reductions in mMASI and hemi‐mMASI scores, confirming the efficacy of MFR for melasma in Fitzpatrick skin types III–IV. The absence of a significant between‐side difference in hemi‐mMASI improvement indicates that, at the level of global clinical pigment scores, adjunctive PRP did not provide an additional benefit over MFR alone [22, 23, 24].

VISIA‐based analysis, however, revealed a more nuanced pattern. Only the combination side showed statistically significant improvements in red area and brown spot scores compared with baseline, whereas corresponding changes on the MFR‐alone side did not reach significance. These findings indicate that the VISIA‐detected improvements on the combination side primarily reflect within‐side changes rather than statistically significant superiority over MFR alone, which is consistent with the absence of between‐side differences in change‐from‐baseline hemi‐MASI scores. In this context, these findings suggest that the addition of PRP may preferentially enhance certain pigmentary and vascular features that are not fully captured by composite clinical indices such as hemi‐mMASI [25, 26]. Given that increased vascularity, low‐grade inflammation, and epidermal hyperpigmentation are key components of melasma pathophysiology, the selective VISIA‐detected benefit on the combination side supports a potential modulatory role of PRP on vascular and inflammatory pathways, particularly in patients with erythematous or mixed‐type melasma [27, 28, 29].

At a mechanistic level, microneedle fractional radiofrequency delivers controlled thermal energy into the dermis through insulated needles, creating focal coagulation zones that trigger neocollagenesis, elastin reorganization, and partial restoration of the basement membrane [30, 31, 32, 33]. These changes may stabilize the epidermal–dermal junction, reduce dermal melanin incontinence, and improve background skin texture, thereby contributing to the observed reductions in mMASI and hemi‐mMASI. PRP provides a concentrated pool of growth factors—such as TGF‐β1, PDGF, and VEGF—which can downregulate melanogenesis‐related signaling, modulate endothelial activity, and attenuate chronic inflammation [25, 26, 27, 28, 29]. It is therefore plausible that PRP mainly augments vascular and fine pigmentary changes captured by VISIA, whereas the stronger structural remodeling induced by MFR dominates the global clinical and ultrasound outcomes [34, 35, 36].

Ultrasound measurements demonstrated significant post‐treatment increases in dermal thickness at selected facial landmarks on both sides, while the magnitude of change did not differ significantly between treatments. This pattern indicates that MFR itself is primarily responsible for the dermal remodeling detectable by ultrasound, and that the addition of PRP did not confer a further measurable effect on structural parameters within the timeframe of this study [30, 31, 32]. The observed dermal thickening is consistent with the established mechanism of MFR: controlled radiofrequency‐induced thermal injury stimulates collagen neosynthesis, elastin reorganization, and stabilization of the epidermal–dermal junction, which may indirectly improve pigment distribution and skin texture in melasma [33, 34, 35, 36].

The lack of a clear additive effect of PRP on mMASI and ultrasound endpoints may be explained by several factors. First, the MFR protocol used here may already achieve a near‐maximal clinical response in terms of global scores, leaving limited room for additional improvement [37, 38, 39, 40]. Second, our PRP preparation and injection schedule, although standardized, may not represent the optimal regimen for melasma; alternative concentrations, delivery depths, or treatment frequencies could potentially yield more pronounced benefits. Third, the modest sample size and follow‐up duration, together with the absence of an a priori power calculation, may have limited the statistical power to detect small between‐side differences, particularly for secondary outcomes [41, 42, 43]. Despite these limitations, this study has several strengths. The randomized split‐face design minimizes inter‐individual variability and allows each patient to serve as their own control, while evaluator blinding reduces the risk of assessment bias. Importantly, we combined conventional clinical scoring with objective tools—VISIA imaging and ultrasound—to provide a multidimensional assessment of treatment response [22, 29]. The inclusion of patients with Fitzpatrick skin types III–IV, who are at increased risk of post‐inflammatory hyperpigmentation with energy‐based devices, further enhances the clinical relevance of our findings. The favorable safety profile observed in this cohort supports the use of MFR as a relatively safe option in darker phototypes [24, 25].

From a practical standpoint, our results suggest that MFR can be considered a core treatment modality for stable facial melasma in Fitzpatrick III–IV patients. The incremental value of PRP appears to be concentrated in VISIA‐detected red area and brown spot parameters, rather than in global pigment scores or ultrasound‐based structural changes [44, 45, 46]. Clinicians should therefore weigh the additional cost and procedural complexity of PRP against its more focused, parameter‐specific benefits when designing individualized treatment plans. Future studies with larger sample sizes, longer follow‐up, and optimized PRP protocols are warranted to determine which patient subgroups derive the greatest benefit from combination therapy and to clarify its impact on long‐term relapse and maintenance strategies [47, 48].

## 5 Conclusions

Our split‐face, randomized trial evaluated the safety and efficacy of microneedle fractional radiofrequency (MFR) with or without platelet‐rich plasma (PRP) for the treatment of facial melasma in Fitzpatrick skin types III–IV. A single course of MFR was safe and effective, with meaningful reductions in mMASI and hemi‐mMASI scores and only mild, transient adverse events. Adding PRP maintained this favorable safety profile and was associated with additional VISIA‐detected improvements in red areas and brown spots, suggesting a useful adjunctive role in patients with erythematous or mixed‐type melasma.

## Author Contributions

Ziyan Chen, Yuhao Li, Yi Ou, Yangmei Chen, and Jin Chen contributed to the study's conception and design. Ziyan Chen and Yuhao Li contributed to the acquisition of data and writing of the initial article. Ziyan Chen, Yi Ou, Yangmei Chen, and Jin Chen performed the statistical analyses and interpretation of data. Ziyan Chen and Tingqiao Chen provided editing support. All authors contributed to the interpretation and analysis of the literature, as well as careful and critical revision and approval of the final manuscript.

## Funding

This work was supported by the Program for Youth Innovation in Future Medicine of Chongqing Medical University, the Chongqing Talent Plan Project (Grant cstc2024ycjhbgzxm0177), and the Chongqing Science and Technology Commission (Grant 2023NSCQ‐MSX0321).

## Ethics Statement

The present study was approved by the Ethics Committee of the First Affiliated Hospital of Chongqing Medical University (approval date: January 8, 2024; clinical trial ethic number: 2024‐079‐01). Written informed consent was obtained from each participant before enrollment, and this study adhered to the Principle of the Declaration of Helsinki.

## Conflicts of Interest

The authors declare no conflicts of interest.

## Data Availability

The datasets used during the current study are available from the corresponding author upon reasonable request.
